# The G*^516^*T *CYP2B6* Germline Polymorphism Affects the Risk of Acute Myeloid Leukemia and Is Associated with Specific Chromosomal Abnormalities

**DOI:** 10.1371/journal.pone.0088879

**Published:** 2014-02-24

**Authors:** Aggeliki Daraki, Sophia Zachaki, Theodora Koromila, Paraskevi Diamantopoulou, Gabriel E. Pantelias, Constantina Sambani, Vasiliki Aleporou, Panagoula Kollia, Kalliopi N. Manola

**Affiliations:** 1 Laboratory of Health Physics, Radiobiology & Cytogenetics, National Centre for Scientific Research (NCSR) “Demokritos”, Athens, Greece; 2 Department of Genetics & Biotechnology, Faculty of Biology, National and Kapodistrian University of Athens, Athens, Greece; NIDCR/NIH, United States of America

## Abstract

The etiology of acute myeloid leukemia (AML) underlies the influence of genetic variants in candidate genes. The CYP2B6 enzyme detoxifies many genotoxic xenobiotics, protecting cells from oxidative damage. The *CYP2B6* gene is subjected to a single-nucleotide polymorphism (G*^516^*T) with heterozygotes (*GT*) and homozygotes (*TT*) presenting decreased enzymatic activity. This case-control study aimed to investigate the association of *CYP2B6* G*^516^*T polymorphism with the susceptibility of AML and its cytogenetic and clinical characteristics. Genotyping was performed on 619 AML patients and 430 healthy individuals using RCR-RFLP and a novel LightSNip assay. The major finding was a statistically higher frequency of the variant genotypes (*GT* and *TT*) in patients compared to the controls (*GT*:38.8% vs 29.8% and *TT*:9.3% vs 5.3% respectively) (*p*<0.001). More specifically, a significantly higher frequency of *GT+TT* genotypes in *de novo* AML patients (46.6%) and an immensely high frequency of *TT* in secondary AML (*s*-AML) (20.5%) were observed. The statistical analysis showed that the variant T allele was approximately 1.5-fold and 2.4-fold higher in *de novo* and s-AML respectively than controls. Concerning FAB subtypes, the T allele presented an almost 2-fold increased in AML-M2. Interestingly, a higher incidence of the *TT* genotype was observed in patients with abnormal karyotypes. In particular, positive correlations of the mutant allele were found in patients carrying specific chromosomal aberrations [-7/del(7q), -5/del(5q), +8, +21 or t(8;21)], complex or monosomal karyotypes. Finally, a strikingly higher frequency of *TT* genotype was also observed in patients stratified to the poor risk group. In conclusion, our results provide evidence for the involvement of the *CYP2B6* polymorphism in AML susceptibility and suggest a possible role of the *CYP2B6* genetic background on the development of specific chromosomal aberrations.

## Introduction

Acute myeloid leukemia (AML) is defined as a clonal proliferation of immature hematopoietic progenitors with varying degree of myeloid differentiation in the bone marrow, peripheral blood, or extra medullary tissues [1]. AML represents the most frequent acute leukemia in adults with a peak of incidence at approximately 65 years, while is more rarely found in children [2]. It constitutes a broad range of disorders with marked clinical and biological heterogeneity. It can be divided in *de novo* AML and secondary AML (*s*-AML) which includes patients with an antecedent hematologic disease such as myelodysplastic syndrome (MDS) or myeloproliferative neoplasm (MPN), or patients with a preceding hematologic or non-hematologic neoplasm treated with chemotherapy and/or irradiation [3]. The AML subtype characterization, crucial for therapeutic decisions, is nowadays based mainly on cytogenetic and molecular alterations of blast cells or on morphology and immunophenotype in the absence of a specific genetic marker [4]. In adult patients, cytogenetic abnormalities are identified in 55–60% of newly diagnosed *de novo* AML and in 85–90% of *s*-AML [5]–[7]. The etiology of the disease is currently unknown; however, the interaction between environmental exposure and genetic susceptibility has been postulated to be a possible cause for the development of AML [8].

Certain detoxification genes which encode antioxidant enzymes, such as *NQO1*, *GSTs* (*GSTT1*, *GSTM1* and *GSTP1)* and cytochrome P450 genes (*CYP2D6*, *CYP1A1*, CYP3A5, *CYP2E1)* are known as AML risk factors [8]–[13]. CYP superfamily comprises phase I detoxification enzymes that metabolise many exogenous and endogenous genotoxic compounds, such as dibenzanthracene, 6-aminochryse, styrene, nicotine and vinylchloride [14]–[18], by insertion of an atom from molecular oxygen into the substrate, actingasmono-oxygenases, oxidases and peroxidises [19]. CYP detoxification enzymes play a key role in protecting cells against oxidative damage. In particular, oxidative stress products are recognised by three crucial cytosolic receptors, namely the pregnane X-receptor (PXR), constitutive androgen receptor (CAR) and aryl hydrocarbon receptor (AhR), which mediate the induction of CYP expression [20]. It has been demonstrated that single nucleotide polymorphisms (SNPs) at the CYP genetic loci inactivate enzymatic activity and may be associated with many types of cancers including haematological malignancies, such as acute lymphoblastic leukemia, myelodysplastic syndromes and acute myeloid leukemia [10], [20]–[24]. The *CYP2B6* gene, a member of the cytochrome P450 superfamily B, is mainly expressed in liver [25]–[27]. To date, more than 100 SNPs resulted in different alleles such as G^516^T, C^64^T, C^777^A, A^785^G, C^1459^T, T^983^C have been characterized at the *CYP2B6* gene locus [28], [29]. Although numerous SNPs have been identified, the G^516^T SNP is the only one that has been associated with leukemia [22], [23].

The G*^516^*T *CYP2B6* genetic variant results in guanine to thymine substitution at nucleotide 516 in exon 4 (rs3745274), and consequently in glutamine to histidine substitution at 172 amino-acid position (Gln172His). This non-sense polymorphism affects metabolic activity by altering substrate binding [30], [31] or aberrant splicing leading to decreased amounts of the normal mRNA transcript and consequently to reduced levels of functional protein [32]. Thus, homozygous individuals for the T allele (*TT*) have a lower enzymatic activity than individuals homozygous for the wild type G allele (*GG*), while heterozygotes *(GT)* display intermediate activity [31], [32]. The frequency of the *CYP2B6* G*^516^*T polymorphism exhibits ethnic variation. In Caucasians, the frequency of the both mutant genotypes (*TT+GT*) is reported to be 21.6%–28.9% [14], [30], [33], while the frequency of the homozygous mutant genotype (*TT*) is limited to 3%–6% [34]–[36]. In Asian population (Chinese, Japanese and Koreans) the prevalence of variant genotypes ranges between 14% and 21% [37]–[39].

Recent studies have shown a strong association between the presence of this inactivating polymorphism not only in hematological malignancies but also in breast cancer [40]. Concerning leukemia, there are only two studies implicating the G^516^T *CYP2B6* polymorphism in myeloid malignancies concerning 36 AML Turkey patients and 164 AML Chinese patients, respectively [22], [23]; no relevant study has been reported in European populations. In those studies, the frequencies of variant *GT* and *TT* genotypes were found to be increased in patients with AML compared to the controls, demonstrating an important role of the T variant allele in AML susceptibility. However, only in one of them [23] the G^516^T *CYP2B6* polymorphism was associated with chromosomal abnormalities and more specifically the recurrent genetic abnormalities restricted to the category of WHO 2008 classification. In that study, the investigated polymorphism was found to be increased in 13 AML patients with AML1-ETO fusion gene indicating an association between the t(8;21) and the presence of the mutant T allele [23].

In the present study, in order to evaluate the potential impact of the G*^516^*T *CYP2B6* polymorphism in AML susceptibility, we studied the distribution of the G*^516^*T *CYP2B6* genotypes and allele frequencies in a large cohort of Greek patients (n = 619) with *de novo* or secondary AML and in healthy individuals (n = 430). The *CYP2B6* genotype was also evaluated in respect to patients’ demographic and clinical characteristics and specific chromosomal abnormalities.

## Materials and Methods

### Study Population

The study included 619 AML patients and 430 healthy individuals. Diagnosis was established in Greek hospitals between 2008 and 2012, based on the WHO requirements with ≥20% bone marrow or peripheral blood blasts, except cases carrying the recurrent cytogenetic abnormalities t(15;17), t(8;21), inv(16) or t(16;16). Among patients, 503 had *de novo* AML and 116 had *s*-AML. Healthy donors were age and sex matched unrelated individuals with a negative history of previous malignancies and normal peripheral blood cell counts. Both cases and controls enrolled in the study came from different areas of Greece, having thus, a homogeneous ethnic background. The project was in accordance with the declaration of Helsinki and the protocol of the study was approved by the Ethical Committee of the NSCR “Demokritos”. Informed consent was provided from all AML patients and donors included in the study.

### Cytogenetic Analysis

Bone marrow (BM) samples were obtained at the time of diagnosis and submitted for cytogenetic analysis at the Cytogenetics Unit of NCSR “Demokritos”. The cytogenetic analysis was performed on trypsin G-banded chromosome preparations, from unstimulated BM cultures. Karyotypes were described according to the International System for Human Cytogenetic Nomenclature (ISCN) 2013 [41]. Cytogenetic analysis was considered successful if a clonal chromosomal abnormality was detected or a minimum of 20 metaphases were analysed. Complex karyotypes were defined as those with at least 3 acquired chromosome aberrations in the absence of cytogenetic abnormalities listed under the WHO category “AML with recurrent genetic abnormalities” [42]. Monosomal karyotypes (MK) were defined by the presence of one single autosomal monosomy in association with at least one additional autosomal monosomy or one structural chromosomal abnormality in the absence of core-binding factor (CBF) AML and AML-M3 [43]. Some representative karyotypes of our Cytogenetic analysis are shown in supplementary materials (Figure S1 in File S1). Patients were stratified according to the review article of Mrozek K and Bloomfield CD [44] into three risk groups; favorable: t(8;21)(q22;q22), t(15;17)(q22;q12-21), inv(16)(p13q22)/t(16;16)(p13;q22); intermediate: normal, t(9;11)(p22;q23), -Y, +8, +11, +13, +21, del(7q), del(9q), del(11q), del(20q) and abnormalities not classified as favourable or unfavourable; poor risk group: inv(3)(q21q26)/t(3;3)(q21;q26), inv(8), t(6;9)(p23;q34), t(6;11)(q27;q23), t(11;19)(q23;p13.1), del(5q), −5, −7 and complex karyotypes.

### Genotype Analysis

#### DNA isolation

Total genomic DNA was extracted from bone marrow cells and/or peripheral blood of patients with AML and from peripheral blood leukocytes of healthy donors using QIAamp DNA Blood Mini Kit (Qiagen, Hilden, Germany) following standard procedures according to the manufacturer’s instructions. Extracted DNA was used as template for the subsequent genotypic analysis.

#### *CYP2B6* G^516^T genotyping

The *CYP2B6* genotype analysis was performed using a conventional PCR-RFLP method for all AML and control samples. Additionally, we genotyped 186 patients and 186 control samples using a novel Real-Time PCR assay. The two analyses provided identical results for all the samples studied by the two methods.

The PCR-RFLPs assay was performed using Taq DNA polymerase (Qiagen, Hilden, Germany) with primers and conditions previously described by Lang et?al. [30]. To distinguish the wild-type from the mutant allele, the PCR products were digested with the restriction enzyme BsrI (New England BioLabs, Beverly, MA). BsrI digestion of wild-type G allele results in three fragments of 241 bp, 268 bp and 17 bp, while digestion of mutant T allele produces two fragments of 509 bp and 17 bp. Digestion patterns were detected by electrophoresis on a 2% (w/v) agarose gel, where an uncut PCR product was included as an internal control (Figure S2 in File S1).

**Figure 1:**
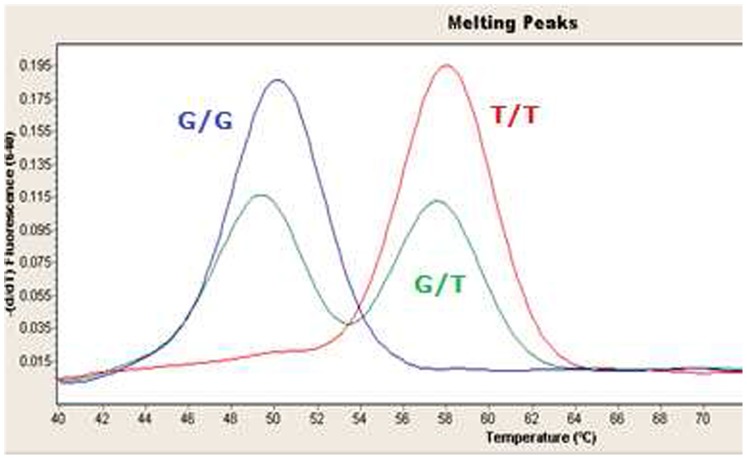
G^516^T *CYP2B6* genotyping by melting curve analysis. Allele-specific derivative melting curve plots for *CYP2B6* G^516^T polymorphism. Fluorescence data were converted to derivative melting curves by plotting the negative derivative of the fluorescence (F) with respect to temperature (T) versus temperature [(dF/dt) vs T] indicating two different melting maxima (Tm), one for the G-allele at 50°C and one for the T-allele at 58°C.

The Real-Time PCR reaction was performed on a LightCycler 2.0 Real-Time PCR System (Roche Diagnostics, Basel, Switzerland), using novel LightSNiP assay (rs3745274), based on SimpleProbe®melting curve analysis (Roche Diagnostics, Basel, Switzerland). To determine melting profiles, sample’s fluorescence decreases during heating, giving that the signal from the probe is quenched as the probe is displaced. The fluorescent data are converted to derivative melting curves by plotting the negative derivative of the fluorescence (F) with respect to temperature (T) versus temperature [-(dF/dT) *vs* T]. Thus, after amplification, the two *CYP2B6* alleles are distinguished by determination of melting curves with the wild type G allele resulted in a melting peak at 50°C and mutant T allele in a melting peak at 58°C (Figure 1).

#### Statistical analysis

For the statistical significance of differences in genotype distributions and allele frequencies between AML patients and controls or among other parameters under study, the Pearson Chi-square test with continuity Yates’ correction was employed. *p*–values less than 0.05 were considered as statistically significant. Odds ratios (ORs) are given with 95% confidence interval (CI). Statistical analysis for multiple testing was performed using the Bonferroni’s correction. Hardy-Weinberg equilibrium was evaluated for our control group using the Chi-squared test. Statistical analysis was performed using SPSS (Statistical Package for the Social Sciences) version 20 software.

## Results

### Clinical and Cytogenetic Characteristics

Table 1 summarizes the clinical characteristics of 619 AML patients and 430 healthy donors evaluated in this case-control study. The patient group comprises 336 males and 283 females (male/female ratio 1.2/1) and control group 239 males and 191 females (male/female ratio 1.3/1). The median age was 61.18 years (range: 16–95) for the AML patients and 64.8 years (range 18–85 years) for the control group. Our patient group consists of 503 *de novo* AML and 116 *s*-AML patients with a median age of 59.5 and 68.9 years at diagnosis, respectively.

**Table 1:** Demographic and cytogenetic characteristics of AML patients and healthy controls.

	AML patients (%)			Controls (%)	
	All cases (n = 619)	de novo (n = 503)	Secondary (n = 116)	n = 430	
**Gender**					
Male	336 (54.3)	280 (55.7)	56 (48.3)	239 (55.6)	
Female	283 (45.7)	223 (44.3)	60 (51.7)	191 (44.4)	
M:F ratio	1.2∶1	1.3∶1	0.9∶1	1.3∶1	
**p-value**	ns				
**Age (yr)**					
Median age	61.18	59.46	68.9		64.8
Range	16–95	16–93	29–95		20–93
**p-value**	ns				
**Age groups**					
<60	263 (42.5)	235 (46.7)	28 (24.2)		190 (44.2)
≥61	356 (57.5)	268 (53.3)	88 (75.8)		240 (55.8)
**p-value**	ns				
**FAB classification** a					
M0	19 (5.7)	19 (5.7)			
M1	28 (8.4)	28 (8.4)			
M2	76 (22.7)	76 (22.7)			
M3	66 (19.8)	66 (19.8)			
M4	79 (23.6)	79 (23.6)			
M5	51 (15.3)	51 (15.3)			
M6	11 (3.3)	11 (3.3)			
M7	4 (1.2)	4 (1.2)			
**p-value**	ns				
**Karyotype** b					
Normal	183 (30.2)	165 (33.7)	18 (15.7)		
Abnormal	422 (69.8)	325 (66.3)	97 (84.3)		
-7/del(7q)	110 (18.2)	77 (15.7)	33 (28.7)		
-5/del(5q)	88 (14.6)	55 (11.2)	33 (28.7)		
+8^c^	59 (9.8)	44 (9.0)	15 (13.1)		
Abn(11q23)	37 (6.1)	32 (6.5)	5 (4.3)		
inv(16)	30 (5.0)	29 (5.9)	1 (1.0)		
t(15;17)	29 (4.8)	29 (5.9)			
t(8;21)	29 (4.8)	28 (5.7)	1 (1.0)		
+21	23 (3.8)	16 (3.3)	7 (6.1)		
-Y	21 (3.2)	21 (4.3)			
t(9;22)	17 (2.8)	10 (2.0)	7 (6.1)		
Other aberrations	87 (14.4)	67 (13.7)	20 (17.4)		
Complex	157 (25.9)	117 (23.9)	40 (34.8)		
Monosomal Karyotypes	118 (19.5)	85 (17.3)	33 (28.6)		
**p-value**	ns				
**Risk group**					
Good	66 (10.9)	64 (13.1)	2 (1.7)		
Intermediate	336 (55.5)	280 (57.1)	56 (48.7)		
Poor	203 (33.6)	146 (29.8)	57 (49.6)		
**p-value**	ns				

*ns: no significance.

a Percentages calculated on the number of de novo AML patients with available FAB classification (334/503).

b Percentages calculated on the number of patients with available cytogenetic data (605/619 AML patients; 490/503 patients with de novo AML and 115/116 patients with s-AML).

c trisomy 8 as a sole chromosomal abnormality, ns: not significant.

The cytogenetic characteristics of AML cases are also summarized in Table 1. Stratification of *de novo* AML patients according to FAB classification showed that the most common FAB subtype was M4 in 23.6% of patients followed by M2 in 22.7%, M3 in 19.8%, M5 in 15.3%, M1 in 8.4%, M0 in 5.7%, M6 in 3.3% and M7 in 1.2%. A successful karyotypic analysis was achieved in 605 out of 619 (97.7%) patients. Among them, abnormal karyotypes were found in 422 (69.8%) patients; 325 with *de novo* AML (66.3%) and 97 with *s*-AML (84.3%). Complex karyotypes were presented in 24.1% of AML patients (21.6% in *de novo* and 34.8% in *s*-AML), while MKs were found in 19.5% of AML patients (17.3% in *de novo* and 28.6% in *s*-AML). The most common chromosomal abnormalities in our cohort of *de novo* AML patients were -7/del(7q) (15.7%), -5/del(5q) (11.2%), isolated +8 (9.0%), abnormalities of 11q23 (6.5%), t(15;17) (5.9%), inv(16) (5.9%), t(8;21) (5.7%) and loss of the Y chromosome (4.3%). As a sole change, loss of the Y chromosome was considered as disease-associated clonal abnormality when it was found in more than 75% of metaphase cells [45]. Similarly, in *s*-AML the most common abnormalities were -7/del(7q) (28.7%), -5/del(5q) (28.7%), t(9;22) (6.1%) and +21 (6.1%). A total of 211 of 605 AML patients carrying more than one recurrent aberration were included in more than one corresponding cytogenetic category. None of patients showed abnormalities on 19q31.2 chromosomal region (*CYP2B6* gene locus) [25]–[27]. In respect to karyotype, our patients were categorized in good (10.9%), intermediate (55.5%) and poor (33.6%) risk groups based on Mrozek K and Bloomfield CD criteria [44].

### *CYP2B6* G^516^T Genotyping and AML Susceptibility

The *CYP2B6* genotypes and allele frequencies in AML patients and healthy individuals are summarized in Table 2. In the control population, the *CYP2B6* genotype was distributed as follows: 64.9% wild-type (*GG*), 29.8% heterozygous (*GT*), and 5.3% homozygous mutants (*TT*). The observed frequencies were in accordance with the Hardy-Weinberg laws of equilibrium (χ^2^ = 2.639 *df* = 1, *p*>0.05), with the homozygous *TT* variant genotype frequency to fall into the range of other Caucasian population studies [35], [36]. The *CYP2B6* genotyping was successfully performed in 572 out of 619 AML patients (92.9%). The genotypic distribution between AML patients and healthy individuals was significantly different, showing a higher frequency of the variant genotypes (heterozygotes *GT* and homozygotes *TT*) in patients compared to the controls (38.8% *vs* 29.8% and 9.3% *vs* 5.3% respectively) (χ^2^ = 17.9, *df* = 2, *p*<0.001). In particular, significantly higher frequencies of the variant genotypes were observed in *de novo* AML patients compared to the controls (χ^2^ = 11.9, *df* = 2, *p* = 0.003). Similarly, increased frequencies of variant genotypes were observed in *s*-AML patients, with the most marked difference to be the immensely high frequency of homozygotes for the mutant allele compared to the controls (20.5% *vs* 5.3%, respectively; χ^2^ = 29.9, *df* = 2, *p*<0.001). It is noteworthy that in 12 out of 618 patients with available BM and PB samples, the genotypic analysis on both samples revealed the same *CYP2B6* genotype.

**Table 2:** Distribution of genotype and allele frequencies of *CYP2B6* G^516^T polymorphism in patients and controls.

Group	*CYP2B6 genotype frequency (%)*				*Allele frequency*			
	*GG*	*GT*	*TT*	*p-value*	*G*	*T*	*p-value*	OR [95% CI]
***Controls (n = 430)***	279 (64.9)	128 (29.8)	23 (5.3)		686 (0.798)	174 (0.202)		
***AML patients*** * ***(n = 572)***	297 (51.9)	222 (38.8)	53 (9.3)	***<0.001***	816 (0.713)	328 (0.287)	***<0.0001***	**1.585** [1.285–1.955]
***de novo AML (n = 465)***	249 (53.6)	185 (39.8)	31 (6.6)	***0.003***	683 (0.734)	247 (0.266)	***0.0016***	**1.426** [1.143–1.778]
***s-AML (n = 107)***	48 (44.9)	37 (34.6)	22 (20.5)	***<0.001***	133 (0.621)	81 (0.379)	***<0.001***	**2.401** [1.739–3.315]

*Genotypic distribution was available in 572 out of 619 samples.

p-value was evaluated after comparison between the *CYP2B6* genotypic distribution of patients and controls.

Distribution of allele frequencies revealed that patients with AML exhibited increased variant T allele frequency compared to the controls (0.287 *vs* 0.202, respectively). Interestingly, the highest T allele frequency was observed in *s*-AML patients (0.379 in *s*-AML *vs* 0.266 *in de novo* AML). The variant T allele was approximately 1.6-fold higher in AML patients (χ^2^ = 18.6, *df* = 1, *p*<0.001, OR = 1.585, 95%CI = [1.285–1.955]) and 2.4-fold in s-AML (χ^2^ = 29.4, *df* = 1, *p*<0.001, OR = 2.401, 95%CI = [1.739–3.315]) than controls.

### Correlation between *CYP2B6* Genotypic Distribution, Clinical and Cytogenetic Characteristics

We also investigated the *CYP2B6* allele frequencies and genotypic distributions in respect to clinical and cytogenetic characteristics. Stratification of patients according to gender revealed an increased variant T allele frequency in females than in males (0.378 *vs* 0.264, respectively; Table S1 in File S1). However, the statistical analysis using multiple testing revealed no significant differences. Furthermore, classification of both patients and controls according to age into two groups (≤60 and ≥61) revealed no statistically significant association concerning the *CYP2B6* genotype and allele frequencies (Table S1 in File S1).

Table 3 summarizes the distribution of the *CYP2B6* genotypes and the allele frequencies in *de novo* AML patients in relation to FAB subtypes. We observed a significantly higher incidence of *CYP2B6* heterozygotes *GT* into the M2 FAB subtype (χ^2^ = 15.4, *df* = 2, *p*<0.001) and homozygotes *TT* into the M6 FAB subtype (χ^2^ = 9.02, *df* = 2, *p* = 0.011) as compared with the control group. Allele frequency distribution analysis confirmed the above differences, showing a higher T allele frequency among AML-M2 and AML-M6 patients (0.329 and 0.500, respectively). The *CYP2B6* variant allele was approximately 2-fold higher in M2 (χ^2^ = 11.6, *df* = 1, *p*<0.001, OR = 1.931, 95%CI = [1.316–2.833]) and an almost 4-fold in M6 than expected (χ^2^ = 6.4, *df* = 1, *p*<0.001, OR = 3.940, 95%CI = [1.256–12.376]). However, the sample size of M6 category was too small to point out any possible association.

**Table 3:** Genotype and allele frequency distribution of *CYP2B6* G^516^T polymorphism in *de novo* AML patients according to FAB classification.

	CYP2B6 genotype frequency (%)				Allele Frequency (%)			
	GG	GT	TT	*p-value	G	T	*p-value	OR [95% CI]
**FAB subtypes^a^**								
**M0** (n = 18)	11 (61.1)	6 (33.3)	1 (5.6)	ns	28 (0.778)	8 (0.222)	ns	
**M1** (n = 22)	13 (59.1)	7 (31.8)	2 (9.1)	ns	33 (0.750)	11 (0.250)	ns	
**M2** (n = 73)	30 (41.1)	38 (52.1)	5 (6.8)	**<0.001**	98 (0.671)	48 (0.329)	**<0.001**	**1.931** [1.316–2.833]
**M3** (n = 65)	40 (61.5)	22 (33.8)	3 (4.7)	ns	102 (0.785)	28 (0.215)	ns	
**M4** (n = 73)	49 (67.1)	19 (26.0)	5 (6.9)	ns	117 (0.801)	29 (0.199)	ns	
**M5** (n = 49)	28 (57.1)	19 (38.8)	2 (4.1)	ns	75 (0.765)	23 (0.235)	ns	
**M6** (n = 6)	2 (33.3)	2 (33.3)	2 (33.3)	**0.011**	6 (0.500)	6 (0.500)	**<0.001**	**3.940** [1.256–12.376]
**p-value**	ns				ns			

ns: no significance.

*p-value was evaluated after comparison with our control population.

The *CYP2B6* genotype and allele frequency distribution in AML patients according to the karyotypic results and the risk group based on cytogenetic findings are presented in Table 4. According to karyotype, an increased frequency of the homozygous variant genotype (*TT*) was observed in patients with abnormal karyotypes, compared to those with normal karyotypes (10.7% *vs* 5.6%, respectively). Allele distribution analysis showed that the *CYP2B6* variant allele was 1.7-fold higher in AML with abnormal karyotypes than our controls (χ^2^ = 21.1, *df* = 1, *p*<0.001, OR = 1.680, 95%CI = [1.346–2.346]). Particularly, the mutant T allele was found to be 2.5-fold higher in *s*-AML patients with abnormal karyotype, and 1.5-fold in *de novo* AML patients with abnormal karyotype than our healthy donors (Table S2, Table S3 in File S1). Based on karyotype complexity, variant *CYP2B6* genotypes presented a borderline significant higher incidence in *s*-AML patients with complex karyotypes (χ^2^ = 5.69, *df* = 2, *p* = 0.056; Table S3 in File S1). Furthermore, in *s*-AML patients presenting monosomal karyotypes, heterozygosity or homozygosity of the mutant T allele was found to be higher (χ^2^ = 5.99, *df* = 2, *p* = 0.050; Table S3 in File S1).

**Table 4:** Genotype distribution and allele frequencies of *CYP2B6* G^516^T polymorphism in AML patients according to karyotype and risk group based on Cytogenetics.

		CYP2B6 genotype frequency (%)				Allele frequency (%)			
	No	GG	GT	TT	p*-value	G	T	*p-value	OR [95% CI]
**Karyotype**									
**Normal**	161	88 (54.7)	64 (39.8)	9 (5.6)	ns	240 (0.745)	82 (0.255)	ns	
**Abnormal**	411	209 (50.9)	158 (38.4)	44 (10.7)	<0.001	576 (0.700)	246 (0.300)	**<0.0001**	**1.680** [1.346–2.346]
**p-value**		ns				ns			
**-7/del(7q)**	109	47 (43.1)	38 (34.9)	24 (22.0)	<0.001	132 (0.606)	86 (0.394)	**<0.0001**	**2.569** [1.868–3.532]
**-5/del(5q)**	86	32 (37.2)	33 (38.4)	21 (24.4)	<0.001	97 (0.564)	75 (0.436)	**<0.0001**	**3.049** [2.161–4.301]
**+8**	58	29 (50.0)	24 (41.4)	5 (8.6)	ns	82 (0.707)	34 (0.293)	**0.025**	**1.635** [1.060–2.521]
**Abn(11q23)**	37	17 (45.9)	19 (51.4)	1 (2.7)	0.024	53 (0.716)	21 (0.284)	ns	
**inv(16)**	27	19 (70.4)	8 (29.6)	0 (0.0)	ns	46 (0.852)	8 (0.148)	ns	
**t(15;17)**	29	15 (51.7)	14 (48.3)	0 (0.0)	ns	44 (0.759)	14 (0.241)	ns	
**t(8;21)**	28	5 (17.9)	21 (75.0)	2 (7.1)	0.001	31 (0.554)	25 (0.446)	**<0.0001**	**3.180** [1.830–5.525]
**+21**	23	9 (39.1)	12 (52.2)	2 (8.7)	0.044	30 (0.652)	16 (0.348)	**0.018**	**2.103** [1.121–3.945]
**t(9;22)**	17	9 (52.9)	6 (35.3)	2 (11.8)	ns	24 (0.706)	10 (0.294)	ns	
**p-value**		**<0.001**				**0.001**			
**Complex**	147	79 (53.8)	55 (37.4)	13 (8.8)	0.042	213 (0.724)	81 (0.276)	**0.009**	**1.499** [1.105–2.034]
**Monosomal Karyotype**	116	60 (51.7)	43 (37.1)	13 (11.2)	0.012	163 (0.703)	69 (0.297)	**0.002**	**1.669** [1.204–2.314]
**Risk group**									
**Good**	63	34 (54)	28 (44.4)	1 (1.6)	0.041	96 (0.762)	30 (0.238)	ns	
**Intermediate**	311	164 (52.7)	130 (41.8)	17 (5.5)	0.003	458 (0.736)	164 (0.264)	**0.005**	**1.412** [1.106–1.802]
**Poor**	198	99 (50)	64 (32.3)	35 (17.7)	0.001	262 (0.662)	134 (0.338)	**<0.001**	**2.017** [1.545–2.632]
**p-value**		**<0.001**				**0.016**			

ns: no significance.

*p-value was evaluated after comparison with our control population.

Further stratification of patients with abnormal karyotypes based on the presence of AML-specific chromosomal aberrations revealed a statistically significant different *CYP2B6* genotypic distribution between the cytogenetic subgroups (χ^2^ = 48.4, *df* = 16, *p*<0.001). This difference was presented mainly due to the increased incidence of homozygous mutant (*TT*) genotypes in patients with -7/del(7q) (24/109, 22.0%) and -5/del(5q) (21/86, 24.4%), as well as heterozygotes (*GT*) in patients carrying trisomy 8 (24/58, 41.4%), trisomy 21 (12/23, 52.2%) and t(8;21) (21/28, 75.0%). The mutant T allele frequencies were 0.446, 0.436, 0.394, 0.348 and 0.293 in patients carrying t(8;21), -5/del(5q), -7/del(7q), +21 and +8 in their karyotype, respectively. Thus, patients showing the abnormalities t(8;21), -5/del(5q), -7/del(7q) have an almost 3-fold increased frequency of carrying the variant T allele compared to the control population (OR = 3.180; 95%CI[1.830–5.525], OR = 3.049; 95%CI[2.161–4.301] and OR = 2.569; 95%CI[1.868–3.532], respectively); patients showing +21 and +8 have an almost 2-fold risk (OR = 2.103; 95%CI[1.121–3.945 and OR = 1.680; 95%CI[1.346–2.346], respectively). The increased frequency of T allele was confirmed in both *de novo* and *s*-AML groups of patients (Table S2, Table S3 in File S1).

As far as it concerns the prognostic groups, a statistically different genotypic distribution (χ^2^ = 27.9, *df* = 4, *p*<0.001) was observed, with a striking higher frequency of the homozygotes for the variant T allele (*TT*) in patients belonging to the poor risk group compared to the other prognostic groups (17.7% *vs* 5.5% in intermediate and 1.6% in good risk group) (Table 4). The allele frequency distribution analysis confirmed these differences, showing a statistically significant higher T allele frequency in the poor prognostic group of AML patients compared to the other risk groups (χ^2^ = 8.24, *df* = 2, *p* = 0.016). Particularly, the mutant allele was 2-fold higher in patients belonging to the poor prognostic group than expected. This finding was confirmed in both *de novo* and secondary AML patients showing an almost 2- fold and 3-fold increased risk of carrying the mutant allele, respectively (Table S2, Table S3 in File S1).

## Discussion

Several reports suggest that genetic predisposition along with exposure in environmental genotoxic compounds may play a pivotal role in the pathogenetic pathways implicated in AML development [24], [46]. *CYP2B6* has been shown to catalyse the oxidation of a number of structurally diverse xenobiotics. Its metabolic activity depends on single-nucleotide polymorphisms (SNPs) [47], such as G*^516^*T germline variation that reduces the enzymatic activity and blocks the biotransformation of carcinogen substrates to harmless metabolites [48]–[50], suggesting that it may be a significant determinant of individual’s risk to AML development. To investigate possible relationships between the *CYP2B6* G*^516^*T polymorphism and AML susceptibility, we performed a case-control study including 572 AML patients and 430 healthy donors. Moreover, the *CYP2B6* polymorphism was also investigated in respect to gender, age, FAB subtype, karyotype and risk classification groups based on cytogenetic findings.

Concerning FAB classification, our patients’ group was found to be comparable to those of prior reports comprising large series of patients with *de novo* AML. Our results revealed that the most common subtype was M4 followed by M2, M3 and M5. According to the literature, M0, M6 and particularly M7 subtypes are consistently rare, whereas the frequencies of the more common subtypes present variability: M1 (range, 16%–27%), M2 (range, 27%–34%), M4 (range, 13%–27%) and M5 (range, 12%–26%) [51]. In our group, M1 and M2 subtypes are lower than the above values. This geographical difference may be random, or can be attributed to the exposure of people in different genotoxic agents or to the different genetic predisposition factors in AML. Moreover, the incidences of clonal chromosomal abnormalities in our patients, are similar to previously reported series [5], [6], [52], [53], with the abnormal karyotypes to be more common in *s*-AML than *de novo.* The stratification of *de novo* AML patients into good (13.1%), intermediate (57.1%) and poor (29.8%) risk groups was found to be comparable to well-defined representative groups of European AML patients [54], [55]. The stratification of *s*-AML patients into the above prognostic groups based only on chromosomal abnormalities revealed an increased frequency of the poor prognostic group (49.6%), which is consistent with the literature [3], [56], [57].

The present study comprises the largest series of AML patients ever evaluated for the G*^516^*T *CYP2B6* gene polymorphism. The observed frequency of the homozygous variant genotype among the Greek healthy donors fell into the range previously reported in Caucasian populations [35], [36]. The major finding was the significantly higher frequency of variant genotypes in AML patients compared to the controls (*p*<0.001). This finding was confirmed for both *de novo* and *s*-AML patients; the variant T allele was found to be 1.5- and 2.4-fold higher in *de novo* and *s*-AML respectively than the control group, indicating a possible effect of the G*^516^*T *CYP2B6* germline polymorphism on AML susceptibility.

The diminished CYP2B6 enzymatic activity, as a result of the presence of the variant T allele, could predispose individuals to be more susceptible to develop AML. This could mean that individuals carrying the variant T allele in homozygous or heterozygous state (*TT* or *GT*) can not efficiently metabolize genotoxic compounds resulting in the accumulation of cell lesions and consequently in the development of AML. According to the above, toxicity related to inefficient detoxification of chemotherapeutic agents used for treatment of a previous hematological malignancy or other cancer, such as cyclophosphamide and ifosfamide [50], [58], [59] may be associated with increased risk of developing s-AML. Our findings are in agreement with the two previous studies evaluated the G*^516^*T *CYP2B6* polymorphism in AML susceptibility and highlighted an increased T allele frequency in AML. In detail, the first study reported a higher frequency of *GT* variant genotype in 36 AML patients from Turkey [22] and the second one a higher frequency of *GT* heterozygotes in 164 AML Chinese patients [23].

Given that AML more commonly affects men than women, we investigated a possible association between gender and *CYP2B6* polymorphism. The weak correlation between the investigated polymorphism and female gender indicates that the presence of the G^516^T polymorphism in females needs further investigation including both genotypic and expression studies. This is because prior expression studies have shown that *CYP2B6* expression is regulated by growth hormone secretion which is sexually dimorphic [60] and females express significantly higher levels of *CYP2B6* compared to males [14]. We also examined possible associations between age and *CYP2B6* polymorphism. No differences were revealed in the frequencies of the *CYP2B6* variant genotypes between patients and healthy donors according to age, suggesting that the *CYP2B6* polymorphism does not modulate AML risk in an age-dependent manner.

Our results showed that the frequencies of the mutant allele and genotypes were significantly higher in patients with M2 and M6 FAB subtypes. In particular, the *CYP2B6* variant allele was approximately 2-fold and 4-fold increased in M2 and M6 patients respectively. However, the finding concerning M6 subtype should be considered with caution, due to the small number of cases. Increased frequencies of the mutant T allele in M2, M1 and M5 AML patients were also found in a recent study [23].

Higher incidence of the variant T allele was observed in AML patients with abnormal karyotype (*p*<0.001); 1.5-fold in *de novo* and 2.5-fold in *s*-AML. Further stratification of patients with abnormal karyotypes according to their chromosomal aberrations showed a higher frequency of *CYP2B6* T allele in AML patients with t(8;21), -5/del(5q), -7/del(7q), +21 and +8. This finding concerns both *de novo* and *s*-AML patients. *CYP2B6* induction by products of oxidative stress in BM comprises a protective mechanism against genetic damage that could contribute to leukemogenesis. Individuals carrying the T allele of *CYP2B6* gene present diminished enzymatic activity and decreased ability to metabolize and inactivate various carcinogens, such as benzene metabolites, alkylating agents, naphthalene, trichloroethylene and aflatoxic B1 [14], [15], [30], [48], [61]. Taken the above together with the increased frequency of variant genotypes observed in AML patients with t(8;21), -5/del(5q), -7/del(7q), +8 and +21, it could be suggested that CYP2B6 enzyme deficiency may affect individual’s vulnerability to hematotoxic exposure to leukemogens and may contribute to an increased risk of AML carrying aberrations of chromosomes 5, 7, 8 and/or 21 and t(8;21). This is strengthened by the increased frequency of trisomy 8 and 21 and deletions of chromosomes 5 and 7 that have been found in peripheral lymphocytes of healthy workers exposed to high concentrations of benzene in a dose-dependent manner [62]–[66]. Moreover, previous occupational exposure to benzene and other organic solvents has been suggested to increase the frequency of aneuploidy of chromosomes 8 and 21 and the translocation between chromosomes 8 and 21 [t(8;21)] [62], [64], [67]. Furthermore, *in?vitro* studies have shown a significant increased frequencies of −5 and −7 in human lymphoblast cell lines after exposure to hydroquinone (HQ) [68], [69]. Therefore, it could be suggested that the *CYP2B6* gene status should be taken into account for treatment optimization with alkylating agents, given that *CYP2B6* deficiency could alter the metabolic capacity against these agents [70] and also that the alkylating factors have been associated with total or partial losses of chromosomes 5 and 7 [71].

Another interesting finding was the higher frequency of the mutant T allele observed in patients with poor prognosis based on cytogenetic findings. This indicates that the presence of the variant allele in homozygous or heterozygous state is probably related with specific chromosomal abnormalities conferring a poor prognosis. Indeed, statistical analysis revealed a strong positive association between the variant *TT* genotype and the presence of the poor prognosis abnormalities -5/del(5q) and/or -7/del(7q) (*p*<0.001). Moreover, *s*-AML patients with monosomal and/or complex karyotypes, known to be related with a poor prognosis, presented also higher frequencies of variant mutant allele as well as variant genotypes. The associations between *CYP2B6* genotype, cytogenetic aberrations, disease course and outcome would be an area of further research.

In conclusion, our results provide evidence for a pathogenetic role of the G^516^T *CYP2B6* polymorphism on AML susceptibility suggesting that inherited defective function of the *CYP2B6* detoxification pathway may be an important genetic determinant of AML risk. The higher frequency of the mutant allele found in patients with specific chromosomal abnormalities or in patients with monosomal and complex karyotypes indicates a strong association between the decreased *CYP2B6* enzymatic activity and the occurrence of certain chromosomal abnormalities in AML. Further studies on this polymorphism in association with the patients’ response to treatment with alkylating agents or inhibitors of topoisomerases II may provide valuable information for the prediction of treatment response in relation to the CYP2B6 genotypes.

## Supporting Information

File S1 **Figures S1 and S2 and Tables S1-S3.** G-banded bone marrow karyotypes of AML patients showing a) 46,XX,del(7)(q22q32) b) 46,XX,del(5)(q13q33) c) 47,XY,+8 d) 45,X,-X,t(8;21)(q22;q22). Figure S2. Gel electrophoresis of G516T CYP2B6 genotyping by BsrI PCR-RFLP on a 2% (w/v) agarose gel. The digestion of the 526-bp PCR product of CYP2B6 yields three bands of 241-bp, 268-bp and 17-bp* for the G/G genotype (wild-type) (lanes 1 and 2), two bands of 509-bp and 17-bp for the T/T homozygous mutant genotype (lane 5), and four bands at 509-bp, 241-bp, 268-bp and 17-bp for the heterozygous G/T genotype (lanes 3 and 4). Lane 6: negative BsrI digestion sample (no target DNA). Lane M: DNA ladder N3236S (New England Biolabs, Inc.). *The small restriction fragment of 17bp is not appeared.
(DOC)
