# Supplementary material for: The G516T CYP2B6 Germline Polymorphism Affects the Risk of Acute Myeloid Leukemia and Is Associated with Specific Chromosomal Abnormalities
Source: PLoS One. 2014 Feb 24;9(2):e88879. doi: 10.1371/journal.pone.0088879 (PMC3933334; doi:10.1371/journal.pone.0088879)
Supplement: File S1 — Figures S1 and S2 and Tables S1-S3. G-banded bone marrow karyotypes of AML patients showing a) 46,XX,del(7)(q22q32) b) 46,XX,del(5)(q13q33) c) 47,XY,+8 d) 45,X,-X,t(8;21)(q22;q22). Figure S2. Gel electrophoresis of G516T CYP2B6 genotyping by BsrI PCR-RFLP on a 2% (w/v) agarose gel. The digestion of the 526-bp PCR product of CYP2B6 yields three bands of 241-bp, 268-bp and 17-bp* for the G/G genotype (wild-type) (lanes 1 and 2), two bands of 509-bp and 17-bp for the T/T homozygous mutant genotype (lane 5), and four bands at 509-bp, 241-bp, 268-bp and 17-bp for the heterozygous G/T genotype (lanes 3 and 4). Lane 6: negative BsrI digestion sample (no target DNA). Lane M: DNA ladder N3236S (New England Biolabs, Inc.). *The small restriction fragment of 17bp is not appeared. (DOC) [file pone.0088879.s001.doc]

Figure S1

a

b


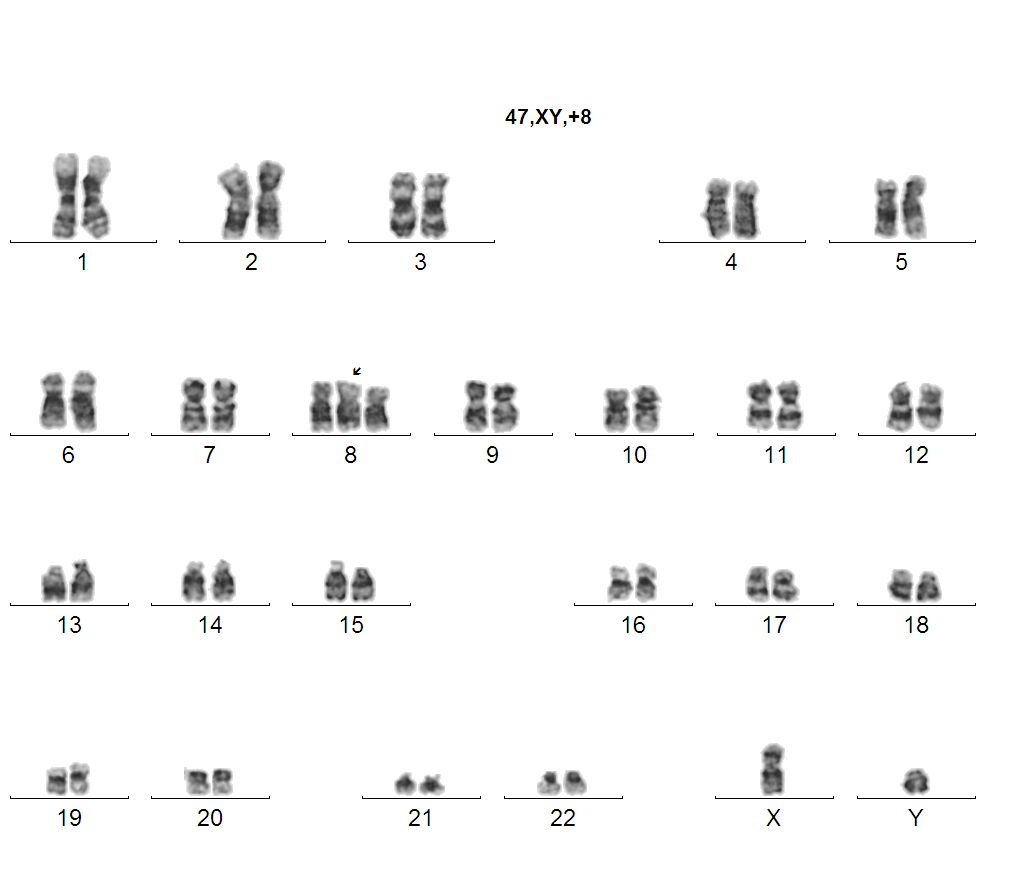


c

d

Figure S2

| **Table S1.** Distribution of *CYP2B6* genotypes in AML patients and control group according to gender and age. | | | | | | |
| --- | --- | --- | --- | --- | --- | --- |
|  |  |  |  |  |  |  |
|  |  | ***CYP2B6 genotype frequency (%)*** | | | ***Allele frequency*** | |
|  | ***No*** | ***GG*** | ***GT*** | ***TT*** | ***G*** | ***T*** |
| ***Gender Control group*** | 430 |  |  |  |  |  |
| Male | 243 | 151 (62.1) | 75 (30.9) | 17 (7.0) | 377 (0.776) | 109 (0.224) |
| Female | 187 | 128 (68.4) | 53 (28.4) | 6 (3.2) | 309 (0.826) | 65 (0.174) |
| *p-*value |  | *ns** | | | *ns* | |
| ***Gender  AML patients*** | 572 |  |  |  |  |  |
| Male | 333 | 185 (55.5) | 120 (36.0) | 28 (8.5) | 490 (0.736) | 176 (0.264) |
| Female | 239 | 112 (46.9) | 102 (42.7) | 25 (10.4) | 326 (0.682) | 152 (0.378) |
| *p-*value |  | *ns* | | | ***0.047*** | |
| ***Age (yr) Control group*** | 430 |  |  |  |  |  |
| ≤60 | 190 | 127 (66.8) | 49 (25.7) | 14 (7.5) | 303 (0.797) | 77 (0.203) |
| ≥61 | 240 | 152 (63.3) | 79 (32.9) | 9 (3.8) | 383 (0.798) | 97 (0.202) |
| *p-*value |  | *ns* | | | *ns* | |
| ***Age (yr)  AML patients*** | 572 |  |  |  |  |  |
| ≤60 | 236 | 128 (54.2) | 81 (34.4) | 27 (11.4) | 337 (0.714) | 135 (0.286) |
| ≥61 | 336 | 169 (50.3) | 141 (42.0) | 26 (7.7) | 479 (0.713) | 193 (0.287) |
| *p-*value |  | *ns* | | | *ns* | |
| * ns: no significance. | | | | | | |

| **Table S2.** Genotype and allele frequency distribution of *CYP2B6* G^516^T polymorphism in *de novo* AML patients according to karyotype and risk groups based on Cytogenetics. | | | | | | | | | | | | | |
| --- | --- | --- | --- | --- | --- | --- | --- | --- | --- | --- | --- | --- | --- |
|  |  |  |  | |  | |  | |  |  | |  |  |
|  |  | ***CYP2B6 genotype frequency (%)*** | | | | | | | ***Allele frequency*** | | | | |
|  | ***No*** | ***GG*** | | ***GT*** | | ***TT*** | | ****p-value*** | ***G*** | | ***T*** | ****p-value*** | ***OR [95%CI]*** |
| ***Karyotype*** | ***465*** |  | |  | |  | |  |  | |  |  |  |
| **Normal** | 146 | 82 (56.2) | | 56 (38.4) | | 8 (5.4) | | *ns* | *220 (0.753)* | | *72 (0.247)* | *ns* |  |
| **Abnormal** | 319 | 167 (52.4) | | 129 (40.4) | | 23 (7.2) | | ***0.003*** | 463 (0.726) | | 175 (0.274) | ***0.001*** | **1.490** [1.171 - 1.895] |
| ***p-value*** |  | *ns* | | | | | |  | *ns* | | |  |  |
| **7/del(7q)** | 77 | 32 (41.5) | | 30 (39.0) | | 15 (19.5) | | ***<0.001*** | *94 (0.610)* | | *60 (0.390)* | ***<0.001*** | **2.516** [1.748 - 3.621] |
| **-5/del(5q)** | 53 | 20 (37.7) | | 21 (39.6) | | 12 (22.7) | | ***<0.001*** | 61 (0.575) | | 45 (0.425) | ***<0.001*** | **2.908** [1.911 - 4.424] |
| **+8** | 43 | 21 (48.8) | | 21 (48.8) | | 1 (2.4) | | ***0.033*** | 63 (0.732) | | 23 (0.268) | *ns* |  |
| **Abn(11q23)** | 32 | 17 (53.1) | | 15 (46.9) | | 0 (0.0) | | *ns* | 49 (0.766) | | 15 (0.234) | *ns* |  |
| **Inv(16)** | 26 | 18 (69.2) | | 8 (30.8) | | 0 (0.0) | | *ns* | 44 (0.846) | | 8 (0.154) | *ns* |  |
| **t(15;17)** | 29 | 15 (51.7) | | 14 (48.3) | | 0 (0.0) | | *ns* | 44 (0.759) | | 14 (0.241) | *ns* |  |
| **t(8;21)** | 28 | 5 (17.9) | | 21 (75.0) | | 2 (7.1) | | ***<0.001*** | 31 (0.554) | | 25 (0.446) | ***<0.001*** | **3.179** [1.829 - 5.524] |
| **+21** | 16 | 8 (50.0) | | 7 (43.8) | | 1 (6.2) | | *ns* | 23 (0.719) | | 9 (0.281) | *ns* |  |
| **t(9;22)** | 10 | 8 (80.0) | | 1 (10.0) | | 1 (10.0) | | *ns* | 17 (0.850) | | 3 (0.150) | *ns* |  |
| ***p-value*** |  | ***<0.001*** | | | | | |  | ***0.001*** | | |  |  |
| **Complex** | 110 | 62 (56.4) | | 39 (35.5) | | 9 (8.1) | | *ns* | 163 (0.741) | | 57 (0.259) | *ns* |  |
| **Monosomal** | 85 | 46 (54.1) | | 30 (35.3) | | 9 (10.6) | | *ns* | 122 (0.718) | | 48 (0.282) | ***0.020*** | **1.551** [1.068 - 2.252] |
| ***Risk group*** |  |  | |  | |  | |  |  | |  |  |  |
| **Good** | 62 | 34 (54.8) | | 27 (43.6) | | 1 (1.6) | | *ns* | 95 (0.766) | | 29 (0.234) | *ns* |  |
| **Intermediate** | 262 | 141 (53.8) | | 111 (42.4) | | 10 (3.8) | | ***0.003*** | 393 (0.750) | | 131 (0.250) | ***0.038*** | **1.314** [1.014 - 1.701] |
| **Poor** | 141 | 74 (52.5) | | 47 (33.3) | | 20 (14.2) | | ***0.001*** | 195 (0.691) | | 87 (0.309) | ***<0.001*** | **1.759** [1.299 - 2.380] |
| ***p-value*** |  | ***0.001*** | | | | | |  | *ns* | | |  |  |
| ns: no significance. | | | | | | | | | | | | | |
| *p-value was evaluated after comparison with our control population. | | | | | | | | | | | | | |

| **Table S3.** Genotype and allele frequency distribution of *CYP2B6* G^516^T polymorphism in *s*-AML patients according to karyotype and risk group  based on Cytogenetics. | | | | | | | | | |
| --- | --- | --- | --- | --- | --- | --- | --- | --- | --- |
|  | | | | | | | | | |
|  |  | ***CYP2B6 genotype frequency (%)*** | | | | ***Allele frequency*** | | | |
|  | ***No.*** | ***GG*** | ***GT*** | ***TT*** | ****p-value*** | ***G*** | ***T*** | ****p-value*** | ***OR [95%CI]*** |
| ***Karyotype*** | **107** |  |  |  |  |  |  |  |  |
| **Normal** | 15 | 6 (40.0) | 8 (53.3) | 1 (6.7) | *ns* | *20 (0.667)* | *10 (0.333)* | *ns* |  |
| **Abnormal** | 92 | 42 (45.7) | 29 (31.5) | 21 (22.8) | ***<0.001*** | 113 (0.614) | 71 (0.386) | ***<0.001*** | **2.477** [1.762 - 3.480] |
| ***p-value*** |  | *ns* | | |  | *ns* | |  |  |
| **-7/del(7q)** | 32 | 15 (46.9) | 8 (25.0) | 9 (28.1) | ***<0.001*** | *38 (0.594)* | *26 (0.406)* | ***<0.001*** | **2.697** [1.594 - 4.564] |
| **-5/del(5q)** | 33 | 12 (36.4) | 12 (36.4) | 9 (27.2) | ***<0.001*** | 36 (0.576) | 28 (0.424) | ***<0.001*** | **3.066** [1.820 - 5.162] |
| **+8** | 15 | 8 (53.3) | 3 (20.0) | 4 (26.7) | ***0.033*** | 19 (0.633) | 11 (0.367) | ***0.029*** | **2.282** [1.066 – 4.885] |
| **Abn(11q23)** | 5 | 0 (0.00) | 4 (80.0) | 1 (20.0) | ***0.010*** | 4 (0.400) | 6 (0.600) | ***0.002*** | **5.913** [1.650 - 21.18] |
| **Inv(16)** | 1 | 1 (100) | 0 (0.0) | 0 (0.0) | *ns* | 2 (1.000) | 0 (0.000) | *ns* |  |
| **+21** | 7 | 1 (14.3) | 5 (71.4) | 1 (14.3) | ***0.021*** | 7 (0.500) | 7 (0.500) | ***0.006*** | **3.943** [1.364 – 11.38] |
| **t(9;22)** | 7 | 1 (14.3) | 5 (71.4) | 1 (14.3) | ***0.021*** | 7 (0.500) | 7 (0.500) | ***0.006*** | **3.943** [1.364 – 11.38] |
| ***p-value*** |  | *ns* | | |  | *ns* | |  |  |
| **Complex** | 37 | 17 (46.0) | 16 (43.2) | 4 (10.8) | ***0.056*** | 50 (0.676) | 24 (0.324) | ***0.014*** | **1.892** [1.131 – 3.165] |
| **Monosomal** | 31 | 14 (45.2) | 13 (41.9) | 4 (12.9) | ***0.050*** | 41 (0.661) | 21 (0.339) | ***0.011*** | **2.019** [3.506 – 3.506] |
| ***Risk group*** |  |  |  |  |  |  |  |  |  |
| **Good** | 1 | 0 (0.0) | 1 (100) | 0 (0.0) | *ns* | 1 (0.500) | 1 (0.500) | *ns* |  |
| **Intermediate** | 49 | 23(46.9) | 19 (38.8) | 7 (14.3) | ***0.011*** | 65 (0.663) | 33 (0.337) | ***0.002*** | **2.002** [1.275 – 3.141] |
| **Poor** | 57 | 25 (43.9) | 17 (29.8) | 15 (26.3) | ***<0.001*** | 67 (0.588) | 47 (0.412) | ***<0.001*** | **2.765** [1.838 – 4.159] |
| ***p-value*** |  | *ns* | | |  | *ns* | |  |  |
| ns: no significance | | | | | | | | | |
| *p-value was evaluated after comparison with our control population. | | | | | | | | | |
